# Flexural strength and microhardness of anterior composites after accelerated aging

**DOI:** 10.4317/jced.53463

**Published:** 2017-03-01

**Authors:** Kanşad Pala, Neslihan Tekçe, Safa Tuncer, Mustafa Demirci, Fatih Öznurhan, Merve Serim

**Affiliations:** 1 DDS, PhD, Assistant Professor, Department of Restorative Dentistry, Faculty of Dentistry, Erciyes University, Kayseri, Turkey; 2 DDS, PhD, Assistant Professor, Department of Restorative Dentistry, Faculty of Dentistry, Kocaeli University, Kocaeli, Turkey; 3 DDS, PhD, Associate Professor, Department of Restorative Dentistry, Faculty of Dentistry, Istanbul University, Istanbul, Turkey; 4 DDS, PhD, Professor, Department of Restorative Dentistry, Faculty of Dentistry, Istanbul University, Istanbul, Turkey; 5 DDS, PhD, Associate Professor, Department of Pediatric Dentistry, Faculty of Dentistry, Cumhuriyet University Sivas/Turkey; 6 DDS, PhD, Department of Restorative Dentistry, Faculty of Dentistry, Kocaeli University, Kocaeli, Turkey

## Abstract

**Background:**

This study aimed to evaluate the flexural strength and microhardness of three different anterior composites after 10 000 thermocycles.

**Material and Methods:**

The mechanical properties of a nano-fill composite (Filtek Ultimate Universal Restorative (FUR) (Enamel)), a nano-hybrid composite (Clearfil Majesty ES2 (ES2) (Enamel)), and a micro-hybrid composite (G Aenial Anterior (GAA)) were investigated in this study. For the microhardness test, 8-mm diameter and 2-mm thickness composite discs were used (n = 10), and for the flexural strength test, 25x2x2 mm bar-shaped specimens were prepared (n = 13). The specimens were tested at 24 h and after 10 000 thermocycles. Data were analyzed using two-way analysis of variance and the post-hoc Tukey test (*p* < .05). Correlations between hardness and flexural strength were calculated using Pearson’s correlation analysis.

**Results:**

There was a significant difference in the microhardness values of the materials (*p* < .05). FUR exhibited significantly higher microhardness than ES2 and GAA. However, the flexural strength of three composites was statistically similar at 24 h (*p* > .05). Pearson correlation analysis revealed that there was a negative relationship between the mean hardness and flexural strength values (correlation coefficient = -0.367, *p* = .043). After 10 000 thermocycles, microhardness values of each material and flexural strength of ES2 and GAA decreased significantly according to 24 h.

**Conclusions:**

The nano-fill composite FUR displayed significantly higher microhardness values. However, each resin composite was statistically similar for flexural strength values. Ten thousand thermocycles significantly affected microhardness and flexural strength.

** Key words:**Flexural strength, microhardness, anterior composites.

## Introduction

Resin composites are valued in restorative procedures of anterior teeth owing to their esthetic, physical, and mechanical properties (1-3). Development in fillers and polymers used in dental composites have allowed for a broad selection of materials that meet the requirements of each clinical situation (4). Anterior composite resin materials used in the restoration of anterior teeth, such as class III or class IV restorations located on incisal edges, must also be very fracture-resistant because high shearing forces cause stress to the restoration during chewing (5). Filler size, shape, and distribution directly determine all of these mechanical and esthetic properties of composites (6,7). Mechanical properties including microhardness and flexural strength are important properties for materials used in restorations where severe biting stress can cause defects, which result in inadequate protection against fracture (8).

Clinical performance and shelf-life claims of new products are often tested using accelerated aging protocols to provide experimental data (9). Water is known to decrease the mechanical properties of the silane interface, cause filler debonding, and degrade resin in resin-based composites (10,11). Most polymer networks are generally considered as insoluble structures with high chemical and thermal stability (12). Nevertheless, these networks can absorb water and chemicals from their environment. Volumetric changes such as swelling, physical changes such as plasticization and softening, and chemical changes can be seen after aging protocols (12).

Thus, the aim of the study was to compare the mechanical properties of three different anterior composites after 10 000 thermocycles. The following null hypotheses were tested: 1) there are no differences in the flexural strength and microhardness values between nano-fill, nano-hybrid, and micro-hybrid composites, 2) there is no relationship between mean hardness and flexural strength values, and 3) 10 000 thermocycles do not affect the flexural strength and microhardness values of anterior composites.

## Material and Methods

In the present study, a nano-fill composite Filtek Ultimate Restorative [(FUR), Enamel, 3M ESPE, St. Paul, MN, USA)], a nano-hybrid universal restorative Clearfil Majesty ES2 [(ES2), Enamel A2 shade, Kuraray Medical Inc., Tokyo, Japan)], and a micro-hybrid composite G Aenial Anterior [(GAA), GC Corporation, Tokyo, Japan)] were compared ( Table 1).

**Table 1:**
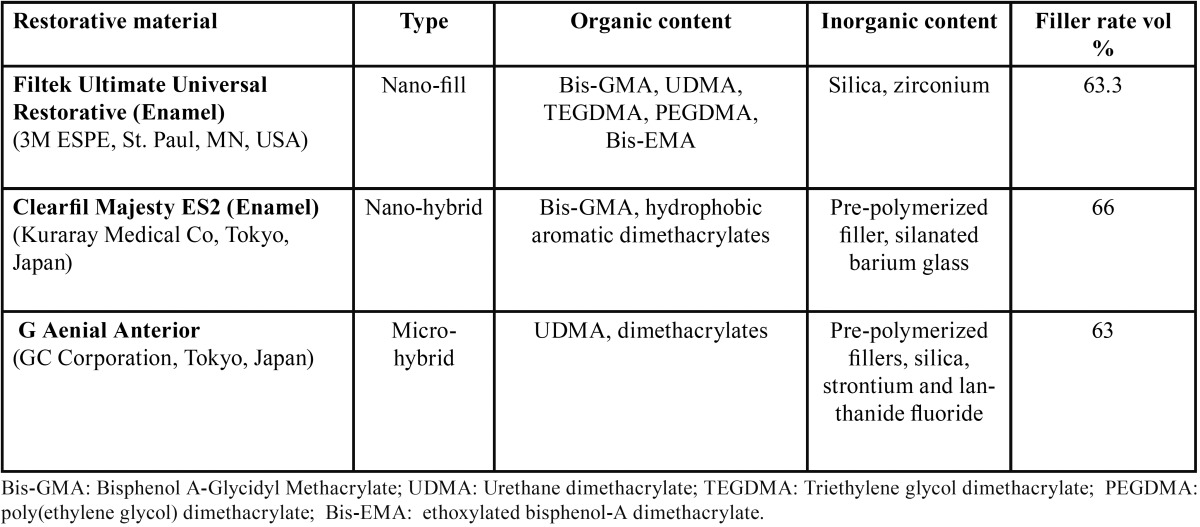
Type, organic, inorganic and filler content of the resin composite materials used in the present study.

-Vickers Hardness Test

In total, 8-mm-diameter and 2-mm-thick 60 circular samples were prepared, 20 of which were FUR, 20 were ES2, and 20 were GAA. Uncured resin composite samples were condensed into a cylindrical stainless steel ring mold in one increment, and the mold was compressed between two glass microscope slides using finger pressure to remove excess resin and ensure a flat surface. The specimens were then polymerized for 40 s using an LED light (Elipar S10, 3M ESPE, St. Paul, USA) at 1200 mW/cm2. Afterwards, all samples were stored in distilled water at 37ºC for 24 h prior to testing. The top surfaces of the specimens were ground with 1200 grit silicon carbide (SiC) paper for 20 s under running water. Finishing and polishing was performed using Sof-Lex disks (3M ESPE, St. Paul, USA).

The Vickers hardness numbers of the specimens were determined at 24 h and after 10 000 thermocycles using a Struers Dura-min-5 microhardness tester (Struers Corp., Tokyo, Japan). Three indentations were pressed into the surface under a 300 g load with a 15 s dwell time. The average hardness value for each specimen was determined for 24-h measurements. Each sample was then submerged in thermocycling and identical measurements were taken after 10 000 thermocycles.

-Flexural strength test

Preparation of fracture strength test specimens was determined in accordance with ISO 4049 (13) by conducting a 3-point bending test on bar-shaped specimens prepared in a 2×2×25-mm-stainless steel mold. In total, 78 circular samples were prepared: FUR (n=26), ES2 (n=26), and GAA (n=26). Half of the specimens from each composite were tested at 24 h and the remaining half were tested after 10 000 thermocycles. Each specimen was photo-polymerized for 20 seconds on both sides in 5 separate overlapping portions using a handheld light-polymerizing unit (Elipar S10, 3M ESPE, St. Paul, MN, USA). The edges of the specimens were manually finished with 1200-grit SIC-paper. The specimens were stored in distilled water at 37ºC for 24 h. The 3-point bend test was then conducted to half of 26 specimens from each composite using a universal material testing machine (LF Plus, LLOYD Instruments, Ametek, Inc., England) under a 0.5 mm/min cross-head speed, span length 20 mm, and a 2-mm-diameter indenter.

-Statistical analysis

Statistical analysis was conducted using the Statistical Package for the Social Sciences v. 20.0 (SPSS, Chicago, IL, USA). Data distribution was checked for normality using the Kolmogorov-Smirnov test. Continuous variables are expressed as mean±standard deviation. Continuous variables for flexural strength and microhardness measurements were compared between the groups using two-way analysis of variance.

## Results

The mean Vickers hardness (kg/mm2), flexural strength (MPa) values, and standard deviations for each resin composite at 24 h and after 10 000 thermocycles are shown in  Table 2.

**Table 2:**
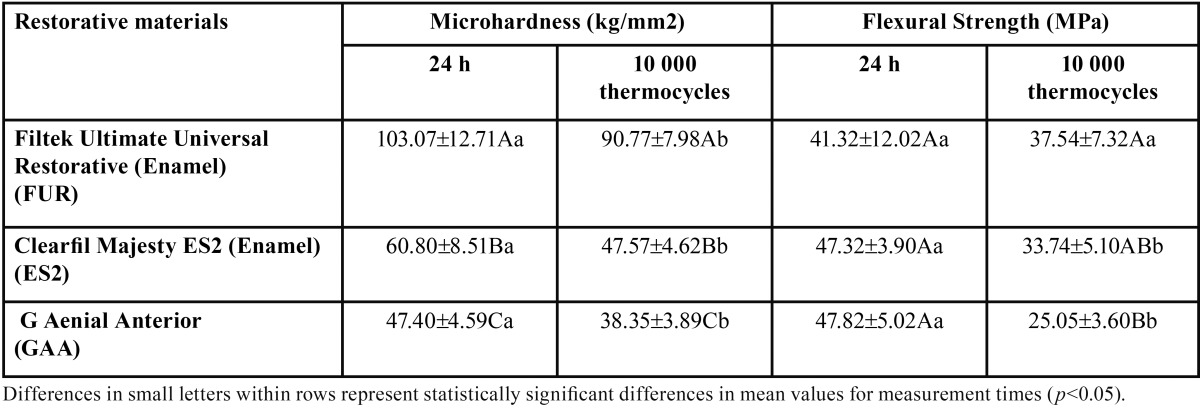
Microhardness (VHN, kg/mm2) (mean ± SD) and flexural strength values (MPa) of the tested materials.

There was a significant difference in the microhardness values of the materials (*p* < .05). FUR (103±12.7 kg/mm2) exhibited significantly higher microhardness values than ES2 (60.8±8.5 kg/mm2) and GAA (47.4±4.6 kg/mm2) (*p* < .05). ES2 displayed significantly higher microhardness values than GAA (*p* < .05). The microhardness value of each composite decreased significantly after 10 000 thermocycles (*p* < .05).

The comparison of flexural strength values of composites revealed that each of the three composites displayed statistically similar results (*p* > .05). However, the micro-hybrid composite GAA (47.82±5.02 MPa) and nano-hybrid composite ES2 (47.3±3.9 MPa) showed slightly higher flexural strength values than the nano-fill composite FUR (41.3±12.2 MPa) (*p* > .05). The flexural strength values of FUR did not change significantly after 10 000 thermocycles (*p* = .104), whereas it decreased significantly for GAA and ES2 (p < .05).

**Figure 1:**
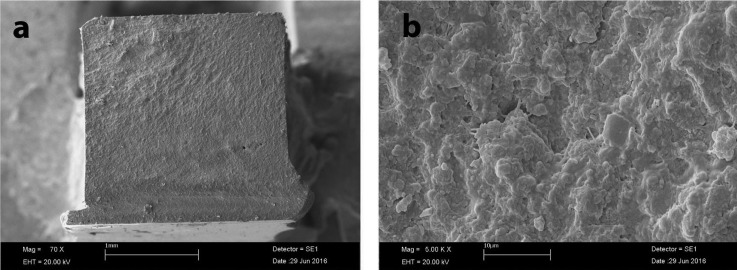
SEM image of fractured specimen of FUR; a: ×70 magnification; b: ×5 00 magnification.

**Figure 2:**
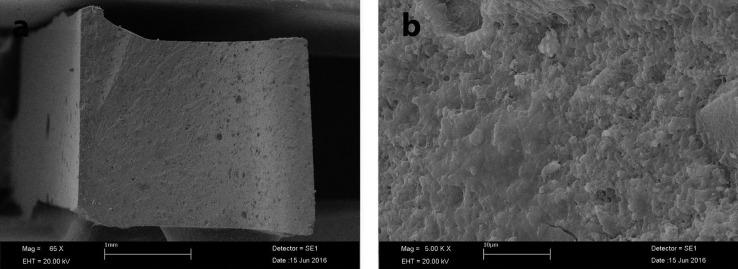
SEM image of fractured specimen of ES2; a: ×70 magnification; b: ×5 00 magnification.

**Figure 3:**
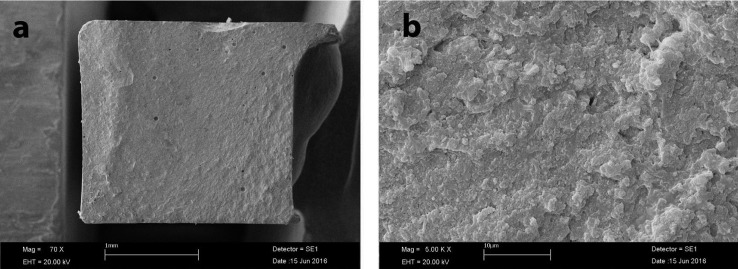
SEM image of fractured specimen of GAA; a: ×70 magnification; b: ×5 00 magnification.

Pearson’s correlation analysis revealed that there was a negative relationship between the mean microhardness and flexural strength values (correlation coefficient = -0.367), (Figs. 1-3).

## Discussion

This study was conducted to investigate the flexural strength and microhardness mechanical behaviors of a nano-fill, nano-hybrid, and micro-hybrid composite, and the influence of thermocycles on the flexural strength and microhardness of these three different anterior composites. It was demonstrated that FUR demonstrated significantly higher microhardness values than GAA and ES2, and ES2 was significantly higher than GAA. On the other hand, micro-hybrid composite GAA, nano-fill FUR, and nano-hybrid ES2 exhibited statistically similar flexural strength values. Therefore, the first null hypothesis that “there is no difference in the flexural strength and microhardness values between the nano-fill, nano-hybrid, and micro-hybrid composites” must be rejected. The second null hypothesis, “there is no relationship between the mean hardness and flexural strength values” must also be rejected, because there was a negative correlation between hardness and flexural strength for the anterior composites. Our third hypothesis, “Ten thousand thermocycles do not affect flexural strength and microhardness values of anterior composites” must be rejected because both parameters changed significantly after 10 000 thermocycles.

The highest microhardness values in the study were obtained with FUR. ES2 and GAA exhibited significantly lower microhardness values than FUR. Composites that include pre-polymerized fillers (ES2 and GAA) exhibited significantly lower microhardness values in the present study. Blackham *et al.* (14) reported that pre-polymerized fillers that contained composites (Gradia Direct Posterior, Premise) performed worse in strength tests than traditional hybrid composites (Z250, Esthet-X). According to Kim *et al.* (15) filler morphology and loading influenced mechanical properties of composites such as flexural strength and microhardness. The researchers reported that pre-polymerized filler particle-containing composites had significantly lower flexural strength compared with other composites. Pre-polymerized resin filler is primarily added into composites to reduce dimensional change during polymerization (16) and to reduce the amount of unpolymerized resin (17). However, use of pre-polymerized filler might result in an actual lower percentage of filler, which may result in poorer mechanical properties. The three different composites used in the present study that were all anterior composites and they incorporate with different combinations of silica, zirconium, barium glass, fluoroaluminosilicate, and pre-polymerized filler; barium glass for radiopacity, amorphous silica for better handling (15). FUR incorporates zirconium particles; higher microhardness values of FUR may be related with zirconia filler. Also, filler distribution or dimensions could affect hardness results (14,18).

In this study, nano-fill, nano-hybrid, and micro-hybrid composites were investigated. Beun *et al.* (19) compared the mechanical properties of nano-filled composites with universal hybrid and micro-filled composites. The authors revealed that micro-filled composites exhibited significantly lower mechanical properties than nano-filled and universal hybrid composites. In addition, nano-filled Grandio and universal hybrid Z 100 had significantly higher microhardness values than those of other composites. Similarly, we found that the micro-hybrid composite GAA showed significantly lower microhardness values than nano-fill FUR and nano-hybrid ES2. One of the results of our study was consistent with a finding of Moraes *et al.* (20) who reported that nano-hybrid resins generally demonstrated inferior properties compared with nano-filled composites, and the behavior of nano-hybrid resin composites was more closely related to that of micro-hybrid than nano-filled materials. Da Silva *et al.* (21) examined the microhardness of nanofilled Filtek Z350 XT, microhybrid Filtek Z250, and microfilled Durafill composites and they found values of 118.40, 123.70, and 39.45 KHN for the composites, respectively. The researchers attributed the lowest values of surface hardness Durafill to it having the highest percentage of organic matrix, based on UDMA, and a lower percentage of inorganic filler. Mohammadi *et al.* (22) examined the microhardness and flexural strength of Silorane and Filtek Z250 composites and found higher flexural strength (114.76 MPa) and microhardness (81.07 Vicker’s hardness) values for Z250 than Silorane, which the authors attributed to the superior mechanical properties of Z250, its higher filler percentage (82%) compared with Silorane (76%).

Flexural strength is considered as the best measure of strength of dental materials and is defined as the maximum stress a material can resist before failure (23); considerable flexural stresses may occur during the complex process of mastication. Heintze *et al.* (24) reported that flexural strength was a good indicator for a material’s durability under stress, and that it correlated well with clinical longevity. Beun *et al.* (19) examined the flexural strength of nine different composites (three nano-fill (Supreme, Grandio and Grandio Flow), four universal hybrid (Point-4, Tetric Ceram, Venus, Z 100), and two micro-fill (A110, Durafill VS) composites, and the authors revealed that all resin composites had similar flexural strength values, with the exception of Durafill VS. Similar to Beun *et al.*, we also found similar flexural strength values for nano-fill, nano-hybrid, and micro-hybrid composites in the present study. In contrast, Hahnel *et al.* (25) found a significant difference between the flexural strength values of their composites (Filtek Supreme XT, Filtek Silorane, CeramX, Quixfil).

In accordance with studies in the literature, thermocycling significantly affected the microhardness of resin composites in the present study. The microhardness values of each material and flexural strength of ES2 and GAA decreased significantly after 10 000 thermocycles compared with 24-h water storage. The decreasing mechanical properties of materials after water storage results from the separation of polymer chains by water molecules (12). Water can cause the degradation of dental composites by weakening the silane interface and leaching filler particles, or softening the organic matrix due hydrolysis. Both effects result in a decrease of mechanical properties of composites. Different from our study, Bauer *et al.* (26) reported that 4-week aging in artificial saliva improved micro-mechanical properties such as Vickers hardness, as compared with 24-h water storage. Hahnel *et al.* (25) investigated the mechanical properties (microhardness and flexural strength) of five different composites after artificial aging protocols (storage in distilled water or artificial saliva or 2×3000 thermal cycles) and revealed that the aging medium had no significant influence on microhardness and flexural strength values. According to Hahnel *et al.* (25) prolonged aging periods (90 or 365 days) or thermocycles led to significant decreases in both microhardness and flexural strength. Göhring *et al.* (27) reported that water storage with and without thermocycling caused deterioration in flexural strength of composites (Bellaglass, Sinfony, Targis) regardless of filler content and resin matrix composition. Janda *et al.* (28) examined the flexural strengths of hybrids, packables, ormocers, compomers, and flowables prior to and after thermocycling. The authors revealed that flexural strength of Solitaire 2 and Admira significantly decreased after 5000 thermocycles. Munksgaard *et al.* (29) reported no significant difference in flexural strength after 180 days of water storage for a traditional (Spectrum) and three different modified resin composites. Cesar *et al.* (30) examined the flexural strength and microhardness values of composites after 30 days of water storage and showed that extended water storage negatively affected the hardness of all composites tested; however, it did not affect the flexural strength of most of the composites. Sideridou *et al.* (31) reported that nanohydrid Grandio (with 71.4 vol% filler loading) showed the high flexural strength values after immersion in water for 30 days than two nanohydrid dental composites (Tetric EvoCeram and Protofill-nano) and two nanofill composites (Filtek Supreme Body and the Filtek Supreme Translucent). The researchers revealed that flexural strength depends on the filler content and also on filler chemistry. Another study of Sideridou (23) *et al.*, reported that the mechanical properties of Bis-GMA showed no significant difference after immersion in water; however, UDMA resin showed a significant decrease between 0 and 30 days for both flexural (73%) and tensile strength (85%). Ho *et al.* (32) reported that UDMA-based materials softened in water much more easily than Bis GMA-based materials. In agreement with Sideridou (23) *et al.* and Ho *et al.* (32), we found microhardness and flexural strength values of UDMA based G Aenial Anterior decreased significantly after thermocycles. The fact that both are UDMA based, and the pre-polymerized filler content of G Aenial Anterior may be responsible for the lower hardness values and stability for GAA.

In another of our studies (33), microhardness values of G Aenial Posterior, Filtek Z550, and Clearfil Majesty Posterior significantly decreased after 10 000 thermocycles. Similarly, in another study (34) we found that microhardness values of Clearfil Majesty Esthetic decreased significantly after one year of water storage and microhardness values of Clearfil Majesty Posterior values were quite stable (138.74-137.25). Clearfil Majesty Posterior was very resistant to aging in water, but significant decreases in microhardness values of Clearfil Majesty Esthetic were observed; we associated the higher stability and high microhardness values of Clearfil Majesty Posterior with its filler content and composition. Indeed, the 92% filler content of Clearfil Majesty Posterior is a very high rate for filler, which would account for the very high hardness values and stability. The results of the decreases in microhardness values of FUR, ES2, and GAA are compatible with the results of other studies because none of the composites in this study had a filler content as high as Clearfil Majesty Posterior.

## Conclusions

FUR demonstrated significantly higher microhardness values than GAA and ES2; ES2 was significantly higher than GAA. On the other hand, the micro-hybrid composite GAA, nano-fill FUR, and nano-hybrid ES2 exhibited statistically similar flexural strength values. There was a negative relationship between the mean hardness and flexural strength values. The microhardness values of each material and flexural strength of ES2 and GAA decreased significantly after 10 000 thermocycles compared with 24-h water storage.
